# Single Nucleus RNA Sequence (snRNAseq) Analysis of the Spectrum of Trophoblast Lineages Generated From Human Pluripotent Stem Cells *in vitro*

**DOI:** 10.3389/fcell.2021.695248

**Published:** 2021-07-21

**Authors:** Teka Khan, Arun S. Seetharam, Jie Zhou, Nathan J. Bivens, Danny J. Schust, Toshihiko Ezashi, Geetu Tuteja, R. Michael Roberts

**Affiliations:** ^1^Christopher S Bond Life Sciences Center, University of Missouri, Columbia, MO, United States; ^2^Division of Animal Sciences, Bond Life Sciences Center, University of Missouri, Columbia, MO, United States; ^3^Department of Ecology, Evolution, and Organismal Biology, Iowa State University, Ames, IA, United States; ^4^Genetics, Development and Cell Biology, Iowa State University, Ames, IA, United States; ^5^Department of Obstetrics and Gynecology, University of Missouri School of Medicine, Columbia, MO, United States; ^6^DNA Core Facility, University of Missouri, Columbia, MO, United States; ^7^Department of Biochemistry, University of Missouri, Columbia, MO, United States

**Keywords:** human embryonic stem cells, BMP4, syncytiotrophoblast, placenta, trophoblast, extravillous trophoblast, snRNASeq

## Abstract

One model to study the emergence of the human trophoblast (TB) has been the exposure of pluripotent stem cells to bone morphogenetic protein 4 (BMP4) in presence of inhibitors of ACTIVIN/TGFB; **A**83–01 and FGF2; **P**D173074 (BAP), which generates a mixture of cytotrophoblast, syncytiotrophoblast, and cells with similarities to extravillous trophoblast. Here, H1 human embryonic stem cells were BAP-exposed under two O_2_ conditions (20% and 5%, respectively). At day 8, single nuclei RNA sequencing was used for transcriptomics analysis, thereby allowing profiling of fragile syncytial structures as well as the more resilient mononucleated cells. Following cluster analysis, two major groupings, one comprised of five (2,4,6,7,8) and the second of three (1,3,5) clusters were evident, all of which displayed recognized TB markers. Of these, two (2 and 3) weakly resembled extravillous trophoblast, two (5 and 6) strongly carried the hallmark transcripts of syncytiotrophoblast, while the remaining five were likely different kinds of mononucleated cytotrophoblast. We suggest that the two populations of nuclei within syncytiotrophoblast may have arisen from fusion events involving two distinct species of precursor cells. The number of differentially expressed genes between O_2_ conditions varied among the clusters, and the number of genes upregulated in cells cultured under 5% O_2_ was highest in syncytiotrophoblast cluster 6. In summary, the BAP model reveals an unexpectedly complex picture of trophoblast lineage emergence that will need to be resolved further in time-course studies.

## Introduction

Early human gestation, especially the approximately 14 days between conception and the anticipated onset of the next menstruation period, is associated with high rates of pregnancy loss (Wilcox et al., 1988). It is the time in pregnancy when the trophoblast (TB) lineage emerges, implantation begins, and a trophoblast interface is established with the mother that allows for physical and nutritional support of the conceptus. Critically, it is also the period when signals from the conceptus trigger the phenomenon of maternal recognition of pregnancy, which prevents the mother from a return to ovarian cyclicity and provides the conceptus some measure of control over maternal physiology and its own future. TB, as the dominant lineage of the emerging placenta, has a major responsibility for all these functions. However, for a range of ethical and practical reasons, these first critical weeks of pregnancy have been largely inaccessible to experimental study. As a consequence, there has been interest in developing models that mimic some of these early events of placental TB emergence *in vitro*.

One approach that has been used to address our poor understanding of these enigmatic early days of pregnancy is extended embryo culture beyond the blastocyst stage (reviewed by Zhou et al. (2021), In Press), made possible because of the availability of “spare” human embryos from *in vitro* fertilization clinics and improved culture media that permitted development until at least day (d) 13 (Deglincerti et al., 2016; Shahbazi et al., 2016). These embryos, although separated from maternal influence, appear to follow a differentiation pathway quite similar to that inferred from the limited number of histological studies performed on *in vivo* material many years before (Hertig et al., 1956; Boyd and Hamilton, 1970). Single-cell RNA sequence (scRNAseq) analysis performed on such embryos at d 8, 10, and 12 of culture revealed the presence of emergent TB populations for syncytioTB and extravillous-like cytoTB that appeared to be similar to, but nonetheless distinct from, TBs that populate the villous placental structures that arise a little later in pregnancy (West et al., 2019). Extended human embryo culture has also provided insights into the mechanisms of TB lineage divergence around the time of implantation (Lv et al., 2019; West et al., 2019; Zhou et al., 2019).

A second model to study the TBs of early pregnancy has been to generate them from pluripotent stem cells, specifically human embryonic stem cells (hESCs) and induced pluripotent stem cells after exposure to BMP4. This pathway of directed differentiation was first reported by Xu et al. (2002) and continues be used extensively, albeit with a range of modifications [Reviewed in Roberts et al. (2018); Horii et al. (2019, 2020)] and especially the exclusion of FGF2 (Das et al., 2007). Among the refinements has been to include two pharmaceutical reagents, the ACTIVIN signaling inhibitor **A**83–01, and the FGF2 signaling inhibitor **P**D1730, so-called **BAP** medium (Amita et al., 2013; Yang et al., 2015). Under this regimen, virtually all cells become positive for the pan-trophoblast marker KRT7 by 48 h. HLA-G positive cells, indicative of extravillousTBs, appear soon after and reach a maximum by d 5–6, while the production of human chorionic gonadotropins (hCG) from syncytioTB begins at about d 5 and increases markedly thereafter. The colonies can be maintained in culture for approximately 8–9 days before regions of syncytium begin to loosen from the Matrigel substratum. The BAP model has allowed different-sized cell types, the largest being syncytium (syncytioTB) and the smallest mononucleated cytoTBs, to be partially characterized and compared (Yabe et al., 2016). Microarray analysis has also been performed on HLA-G positive cells isolated on magnetic beads, which demonstrated a resemblance in terms of its markers to extravillousTB derived from placenta (Telugu et al., 2013).

While microarray and RNAseq data from hESC colonies differentiated in response to BMP4 have shown the differentiated cells to be comprised only of TB, with no evidence for contributions from the main germ line lineages, particularly mesoderm (Schulz et al., 2008; Marchand et al., 2011; Telugu et al., 2013; Yabe et al., 2016; Jain et al., 2017), it has become clear that they displayed a different transcriptome profile from primary TBs isolated from the villous placenta. This realization led us to hypothesize that BAP best modeled the primitive placental structure that forms when the blastocyst first implants and establishes residency in the uterine wall. Although the original microarray and RNAseq studies were informatory about the general nature of the TB that formed in response to directed differentiation, they provided little detail about the identity of the different sub-lineages that formed, their relationships to each other, and how they originated. Here we have employed a single nuclei RNAseq approach, which allowed an analysis of fragile syncytioTB structures as well as the more resilient cytoTBs, to display an unexpected heterogeneity of TB sub-lineages in d 8 cultures.

## Materials and Methods

### Human Embryonic Stem Cell Culture and Trophoblast Differentiation

Human ESCs (H1; WA01) originated from WiCell Research Institute. They were cultured and maintained in Matrigel (BD Bioscience)-coated 6-well tissue culture plates (Thermo Scientific) on mTeSR1 medium (Stemcell Technologies) under two different O_2_ concentration conditions, i.e., 20% and 5% at 37°C, as described previously (Yabe et al., 2016). The medium was replaced daily and cells (50,000/well) passaged approximately every 5–6 days. The protocol first described by Amita et al. (2013) was used to drive differentiation of the ESC to TB. Briefly, the mTeSR1 culture medium, which contains a high concentration of FGF2 (100 ng/ml), was replaced with DMEM/F12 medium (Thermo Scientific) containing knock-out serum replacement (KOSR, Invitrogen) and low FGF2 (4 ng/ml) that had been conditioned by mouse embryonic fibroblast (MEFs) (Supplementary Figure 1). After 24 hours, the conditioned medium was replaced with daily changes of DMEM/F12/KOSR medium minus FGF2 but containing BMP4 (10 ng/ml), A83-01 (1 μM), and PD1730740 (0.1 μM), so-called BAP treatment, for 3 days and then the same medium without BMP4 for the following 4 days (AP treatment) (Supplementary Figure 1A). The content of hCG in the culture medium was measured on d 7 and d 8 by ELISA as described by Amita et al. (2013). The hCG concentration was normalized to 10^5^ nuclei for each replicate cultured under the 20% O_2_ and 5% O_2_ conditions. One-way ANOVA was applied by using GraphPad Prism.

### Nuclei Isolation

On d 8 of differentiation, each culture well was rinsed twice with DMEM/F12 and the colonies were partially dispersed by using gentle cell disassociation reagent (GDR) (Stemcell Technologies) (0.6 ml/well; 7 min at 37°C). The colonies were fully dislodged from the substratum by gentle scraping, and nuclei were isolated from the disassociated cells by the protocol provided by 10X Genomics with minor modifications (10x Genomics, 2019). Briefly, 1 ml of chilled lysis buffer (10 mM Tris-HCl, pH 7.4; 10 mM NaCl; 3 mM MgCl_2_; and 0.1% Non-idet^TM^ P40 Substitute in Nuclease-Free Water) was added to each well through a wide-bore pipet tip. After gently pipetting several times, the suspension was kept on ice for 5 min to achieve maximum lysis of cells and centrifuged (500 × *g*: 5 min at 4°C). The pellet was resuspended in 1 ml of nuclear wash and resuspension buffer (NWRB) (1X PBS with 1.0% w/v bovine serum albumin and 0.2 U/μl RNase inhibitor), cell debris were removed by filtration through a 40 μm Nylon cell strainer (Falcon), and the solution was recentrifuged as above. The supernatant solution was removed, and the final nuclear pellet was suspended in 1 ml of NWRB. The complete workflow is documented in Supplementary Figure 1.

### Single Nuclear 3′ RNA-Seq Library Preparation

Libraries were constructed by following the manufacturer’s (10x Genomics, 2019) protocol with reagents supplied in the 10x Genomics Chromium Next Gel Bead-in-Emulsion (GEMs) Single Cell 3′ Kit v3.1. Briefly, the concentrations of nuclei and intact cells in the nuclear preparation were measured with an Invitrogen Countess II automated cell counter. An aliquot of the nuclear suspension (1,000 nuclei per microliter), reverse transcription master mix, and partitioning oil were loaded on a Chromium Next GEM G chip with a nuclear capture target of 5,000 nuclei per library. Post-Chromium controller GEMs were transferred to a PCR strip tube, and reverse transcription was performed on an Applied Biosystems Veriti thermal cycler at 53°C for 45 min. cDNA was amplified for 14 cycles and purified by using Axygen AxyPrep MagPCR Clean-up beads. cDNA fragmentation, end-repair, A-tailing, and ligation of sequencing adaptors was performed according to manufacturer specifications. The final library was quantified with the Qubit HS DNA kit, and the fragment size was analyzed by means of an Agilent Fragment Analyzer system. Libraries were pooled and sequenced on an Illumina NovaSeq S1 flow cell with a goal to generate 50,000 reads per nucleus with a sequencing configuration of 28 base pair (bp) on read1 and 91 bp on read 2 using unique dual indexes.

### Single-Nuclei Sequencing Analysis

The University of Missouri Informatics Research Core Facility pre-processed the data with the 10XGenomics/CellRanger software (v4.0.0) (Zheng et al., 2017), including demultiplexing, fastq file generation, read alignment, and UMI quantification. CellRanger was run with default options against the ENSEMBL GRCh38 reference genome (Schneider et al., 2017) with both pre-mRNA and mRNA feature files. The data were then processed by means of Seurat (v3.2.2) (Stuart et al., 2019), following the recommended practices for scRNAseq data with replicates. All steps used default options unless noted otherwise. Briefly, an expression matrix (count table) containing Unique Molecular Identifiers (UMIs) per nucleus per gene was imported for each replicate as the 10× data object. Only nuclei with greater than 200 but less than 7,500 genes and less than 15% of genes originating from mitochondrial sources were retained. Once the nuclei were quality filtered, the data were imported as a Seurat object, and all mitochondrial and ribosomal protein-coding genes were removed from the expression matrix. The replicates for each condition were integrated by using the FindIntegrationAnchors and IntegrateData commands. The count matrix was scaled and normalized by variance stabilizing transformation (VST) with Seurat’s ScaleData and NormalizeData commands, respectively. The 2,000 most variable features were then selected with the FindVariableFeatures command for the Principle Component Analyses (PCA), which was performed by the RunPCA command. The PCs generated by the PCA were assessed with ElbowPlot and JackStraw analyses by using up to 20 different components. The resulting PCs were used for Jaccard-weighted, shared nearest neighbor (SNN) distance calculations and graph generation. The graph was then subjected to Louvain clustering and Uniform Manifold Approximation and Projection (UMAP) for dimension reduction in order to visualize nuclear transcriptomic profiles in two-dimensional space. After changing the default assay to RNA in the Seurat object, FindMarkers was run to determine the marker genes (genes with a fold change of greater than or equal to 1.5 with an adjusted p-value of less than 0.05) in each cluster by comparing each gene’s expression level against other clusters.

Cluster identities were approximated by using single-cell RNA-Seq data from first trimester human placenta (Vento-Tormo et al., 2018). We used cell-type specific genes as described in PlacentaCellEnrich (Jain and Tuteja, 2021) within the TissueEnrich Bioconductor package (Jain and Tuteja, 2019).

To perform differential expression analysis between O_2_ conditions, metadata in the Seurat object was updated with a new column containing both information about the cluster and the condition. This column was set as Seurat objects identity, and differential expression analyses were performed by using the FindMarkers command. No down-sampling of any cluster was performed since the corresponding clusters had roughly the same number of cells. Genes were considered upregulated in a condition if the fold change was greater than or equal to 1.5, and the adjusted p-value was less than 0.05.

The GitHub repository documenting all the analyses steps is available at https://github.com/Tuteja-Lab/BAP.hESC.d8_snRNAseq. Sequencing data have been deposited in the Gene Expression Omnibus under accession ID GSE171768.

## Results

### Heterogeneity of Cell Types

Exposure of either human embryonic stem cells or human induced pluripotent cells to BAP conditions for five days or more results in the initiation of syncytioTB formation evident as ruffled areas within the colonies and a steep increase in *CGA* and *CGB* expression and hCG production over subsequent days (Amita et al., 2013; Yabe et al., 2016). As previously described, these cells show downregulation of pluripotency genes, and upregulation of trophoblast marker genes compared to undifferentiated cells (Supplementary Table 1). CGA-expressing TBs are known to be confined to what has previously been demonstrated to be the syncytial patches, with little or no staining outside these areas (Das et al., 2007; Schulz et al., 2008; Amita et al., 2013; Yang et al., 2015), while other trophoblast markers, such as HLA-G, are confined to areas outside the syncytium. The heterogeneity of cell types is further demonstrable by the heterogeneity of staining within colonies and differences in intensities of staining for particular trophoblast markers. Together, these data indicate that snRNAseq analysis might be a fruitful approach to examining lineage divergence and cell type diversity.

### Isolation of Nuclei

H1 ESCs were acclimated to 20% and 5% O_2_ conditions prior to BAP-directed differentiation to TB (Supplementary Figure 1A). Sufficient hCG was released into the medium to be detected after d 5. By d7 and d8 copious amounts of hCG were being produced by cultures grown under both O_2_ conditions (Supplementary Figure 1B), although previous studies had detected a significant lag between d6 and d8 under low O_2_, possibly reflective of a slower rate of differentiation than at 20% (Schulz et al., 2008; Amita et al., 2013). At d 8, the colonies were dissociated as completely as possible without destroying syncytial clumps, and nuclei were isolated (Supplementary Figure 1D). This experiment was repeated a second time, also under the same two O_2_ conditions, in order to provide two replicates for each treatment group (Supplementary Figure 1A). The concentrations of nuclei used to prepare libraries for subsequent snRNAseq analysis were comparable, and the content of intact cells in all four preparations was 5% or less (Supplementary Figure 1F).

### snRNA-seq Analysis of Trophoblast Cells

After sequencing and data processing, nuclei were retained for analysis if they had between 200 and 7,500 unique genes detected, and less than 15% mitochondrial reads (Supplementary Figure 2 and Supplementary Table 2). With the possible exception of the first replicate from cultures under 20% O_2_ conditions, the majority of mitochondrial contamination was associated with nuclei that contained a relatively low number of nuclear transcripts (Supplementary Figure 3). From the four preparations, this resulted in a total of 5,355 nuclei passing quality control filters (Supplementary Table 2). In addition to transcripts from mitochondrial genes, sequencing also revealed, as expected, the presence of ribosomal subunit RNAs in all nuclei. Accordingly, all data were filtered to remove mitochondrial and ribosomal RNA transcripts before further analysis with Seurat software.

**FIGURE 1:**
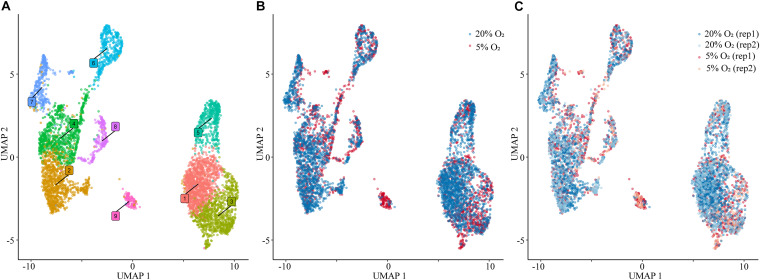
Single nuclear RNA sequencing. **(A)** Visualization of nuclei included in the analysis, colored according to assignment by clustering analysis. **(B)** Plot of the nuclei obtained from the cells cultured under 20% O_2_ (blue) and 5% O_2_ (red) concentration conditions. **(C)** Nuclei plot obtained from each replicate of cells cultured under 20% O_2_ (blue) and 5% O_2_ (red) conditions. For all panels: 5,355 total nuclei were plotted in two dimensions based on transcriptome similarity using uniform manifold approximation and projection (UMAP) for all of the analyses using Seurat. Each dot represents an individual nuclei. 20% O_2_, nuclei obtained from the cells cultured under 20% O_2_; 5% O_2_, nuclei obtained from the cells cultured under 5% O_2_; rep, replicate.

**FIGURE 2:**
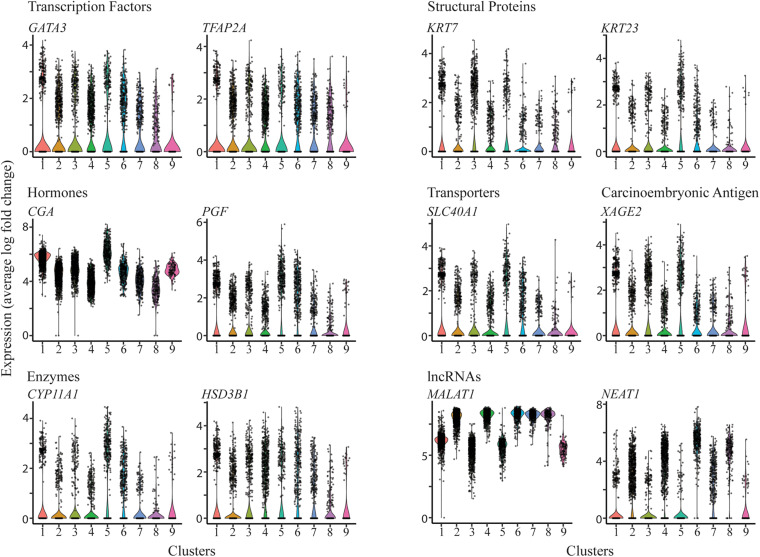
Expression profiles of selected genes across the clusters. Violin plots showing expression for genes encoding transcription factors (**upper left panel**), structural proteins (**upper right panel**), hormones (**mid left panel**), transporters and carcinoembryonic antigen (**mid right panels**), enzymes involved in steroid biosynthesis (**lower left panel**), and long non-coding RNAs (**lower right panel**).

Following cluster analysis and projection of data in uniform manifold approximation and projection (UMAP) expression space, two major groupings, one comprised of five (2,4,6,7,8) and the second of three (1,3,5) clusters, were evident (Figure 1). Cluster 9 appeared to be separated from these two groupings. The gene expression signatures that distinguished the clusters are shown in Supplementary Table 3 and illustrated graphically for each cluster in Supplementary Figure 4. The four different nuclear preparations replicated each other well (Figure 1B). Cluster 9 was again the exception and was comprised almost entirely by contributions from cells differentiated under 5% O_2_. All nine clusters contained nuclei that expressed genes that have traditionally been used in various combinations as markers for TB. These genes included, among others, ones encoding transcription factors, e.g., *GATA3, TFAP2A*, structural proteins, e.g., *KRT7, KRT23*, hormones, e.g., *CGA, PGF*, transporters, e.g., *SLC40A1*, the carcinoembryonic antigen XAGE2, and enzymes involved in steroid biosynthesis, e.g., *CYP11A1, HSD3B1*. However, it is also clear that the expression of any particular gene was variable between individual nuclei within clusters and that mean expression values differed across clusters (Figure 2). In addition, all clusters had high levels of the placenta-enriched, long non-coding RNAs, *MALAT1* and *NEAT1* (Figure 2). Both of the latter were expressed most robustly in the grouping on the left (a) comprised of clusters 2, 4, 6, 7, and 8 compared to the right grouping (b) (clusters 1, 3, and 5) (Figure 2).

There was no significant expression of most marker genes examined for mesoderm, including *DLL3, FOXC1, RIPPLY, T/BRA, FOXA2, MIXL1*, endoderm, including *AFP, GATA4, GDF1, GDF3, MIXL2*, and ectoderm, including *FGF5, OTX2, SOX1*, *PAX6*. A few such marker genes, e.g., *TWIST2, GATA6, NES*, were detected at low levels (Supplementary Figure 5), but at least one of them (*TWIST2*) has a previously described functional association with TB (Ng et al., 2012). These data are generally consistent with the view expressed previously that the BAP-driven conversion of ESCs to TBs is largely, if not entirely, complete and leads to little or no differentiation of the ESCs along the main germline lineages (Amita et al., 2013; Yabe et al., 2016; Roberts et al., 2018).

### Trophoblast Nature of the Clusters

**FIGURE 3:**
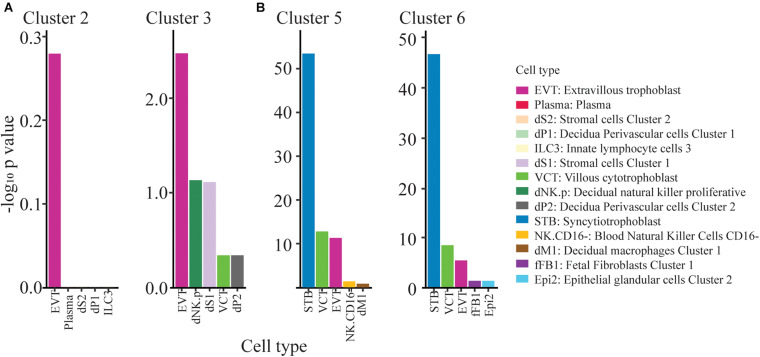
ExtravillousTB and syncytioTB gene enriched clusters. **(A)** Clusters 2 and 3 show weak enrichment for ExtravillousTB specific markers. **(B)** Clusters 5 and 6 show strong enrichment for SyncytioTB specific markers. Top 5 cell type results from PlacentaCellEnrich have been shown here for clusters 2,3,5, and 6. The y-axis corresponds to −log_10_ of the adjusted p-value.

The question then arose as to the kind of TB represented in clusters 1–9. We used the PlacentaCellEnrich program (Jain and Tuteja, 2021) to determine if marker genes from clusters 1-9 were enriched for genes with cell-type specific expression in first trimester placenta, according to data from Vento-Tormo et al. (2018). This analysis provided strong evidence that clusters 5 and 6, were enriched for nuclei with a profile similar to that of placental syncytioTB, while cluster 3 embodied some features of extravillousTB from first trimester placenta (Figure 3). Cluster 2 showed most similarity to extravillousTB, although the adjusted P value when compared to the scRNAseq analysis of first trimester placenta conducted by Vento-Tormo et al. (2018) was not significant indicating that the similarities were low and therefore the cluster identity is unclear. Data for the remaining clusters 1, 4, 7, 8, and 9 were more equivocal, as they were not dominated by a particular set of TB markers, but rather expressed a majority of them (Figure 2). They may represent less well-differentiated cytoTB populations.

**FIGURE 4:**
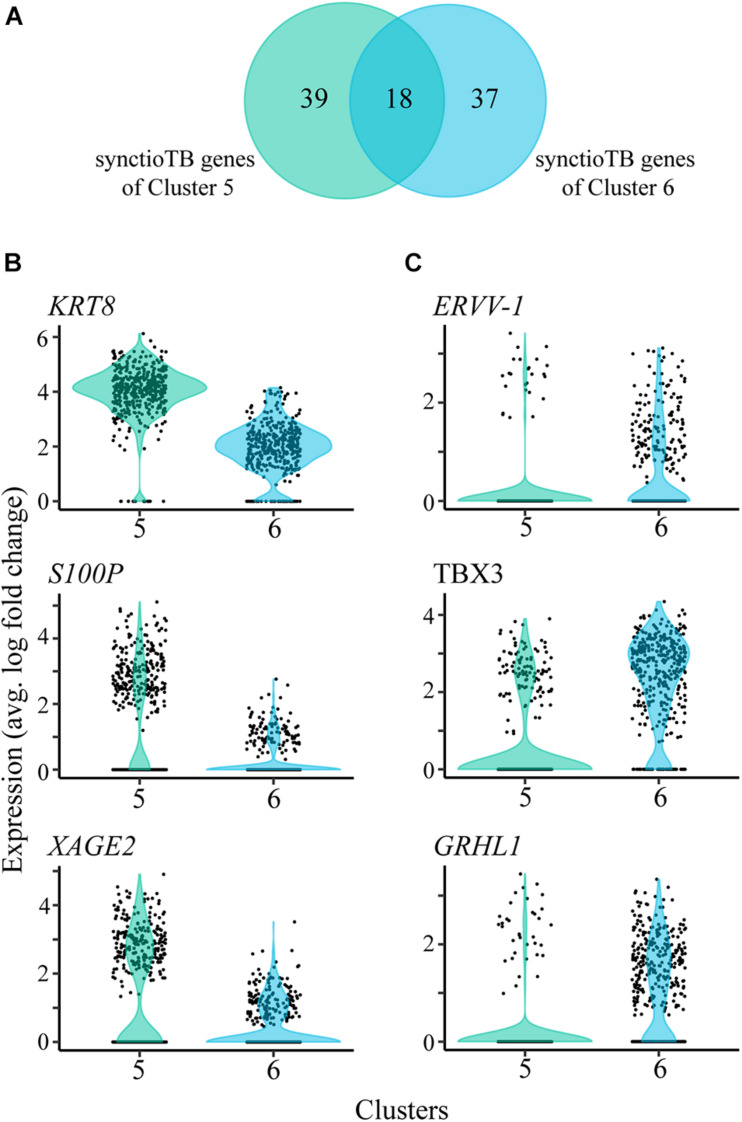
SyncytioTB-specific gene expression profiles. **(A)** Comparison of the SyncytioTB specific genes in clusters 5 and 6. The two clusters have many differentially expressed genes, with some that are more highly expressed in cluster 5 **(B)** and others that are more highly expressed in cluster 6 **(C)**.

Clusters 5 and 6, which carry the more definitive hallmark features of placental syncytioTB, occupy the b and a cluster grouping (Figure 1A), respectively. However, they also differ markedly in the expression of several genes considered to be highly expressed in placental syncytioTB (Figure 4A, Supplementary Table 4). Of the 57 genes labeled as syncytioTB-specific in cluster 5 and the 55 genes labeled as syncytioTB-specific in cluster 6, only 18 were in common. Examples of genes up-regulated in cluster 5 compared to cluster 6 include *KRT8, S100P*, and *XAGE2* (Figure 4B). The reverse was observed for *ERVV-1*, and *TBX3* (Figure 4C), for example. A second endogenous retroviral gene, *ERVW-1*, had much higher expression in cluster 6 than in cluster 5 (expression values 12.76 versus 4.27, respectively). In general, more transcription factors were identified with higher expression in cluster 6 (44 transcription factors) compared to cluster 5 (15 transcription factors).

Cluster 2 (in the major grouping a; Figure 1A) and Cluster 3 (group b; Figure 1A) each showed most similarity to first trimester human placental extravillousTB. Although the similarity was weak, the clusters also lacked indications of a major contribution of other TB cell types (Figure 3). The two are also clearly distinct from each other (Figure 1A). Among the transcripts that distinguish Cluster 2 from 3 are ones that encode TLE4, a transcriptional co-repressor that regulates WNT-mediated beta-catenin signaling, the procadherin PCDH9, and MAML2, a co-activator that binds to the intracellular domain of NOTCH receptors (Supplementary Figure 6). Cluster 3 noticeably possesses a group of upregulated genes that are overexpressed relative to Cluster 2 and whose functions are linked to the structure and organization of the cytoskeleton. Among these are *ACTG1, TMSB10*, and *TAGLN*, as well as three calcium binding proteins (*S100A11, S100A6*, and *S100A10*) (Supplementary Figure 6).

### Effects of O_2_ Atmosphere

**FIGURE 5:**
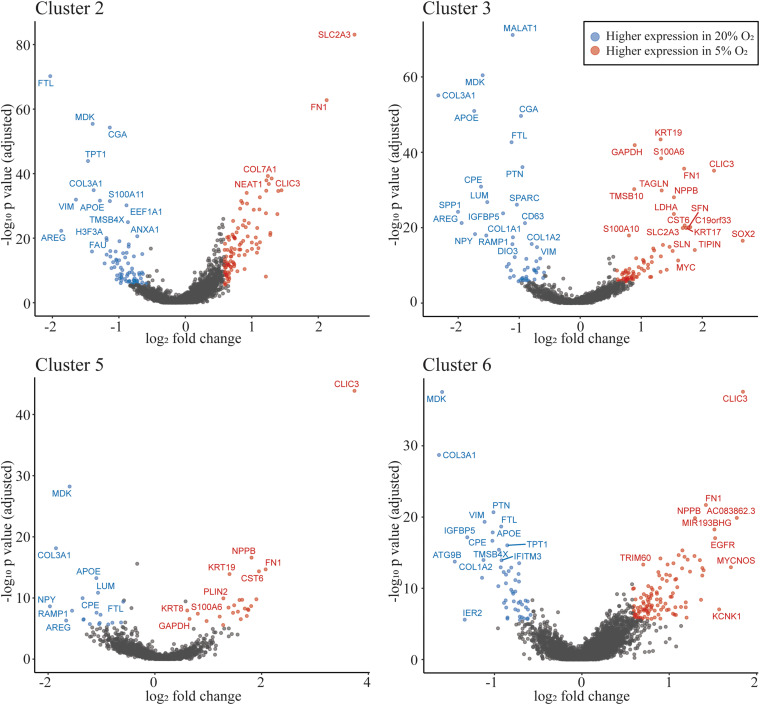
Oxygen concentration influences gene expression profiles. Volcano plot showing differentially expressed genes in clusters 2, 3 (most similar to extravillousTB), 5 and 6 (most similar to syncytioTB). The highlighted dots show genes with an absolute fold change ≥1.5, with an adjusted p-value of ≤0.05. The genes shown as dots in blue have higher expression in 20% O_2_ and red dots have higher expression in 5% O_2_.

We identified differentially expressed genes between oxygen conditions in clusters 1–8. Cluster 9 is dominated by nuclei from 5% O_2_, which did not allow evaluation of differentially expressed genes. The number of differentially expressed genes for clusters 1–8 ranged from 37 to 188 (Supplementary Table 5). Of the TB clusters with most similarity to extravillousTB (cluster 2 and cluster 3), and the TB clusters predicted to be syncytioTB (cluster 5 and cluster 6), cluster 5 has the least number of differentially expressed genes (Supplementary Table 5). However, all of these clusters include genes associated with metabolism that were up-regulated in the 5% O_2_ cultures (SLC2A3). Additionally, there are several other up-regulated transcripts in common in clusters 2,3,5, and 6, such as *CLIC3* and *FN1* (up-regulated in the 5% O_2_ cultures) and *APOE*, *COL3A1*, and *LUM* (up-regulated in the 20% O_2_ cultures; Figure 5, Supplementary Figure 7, Supplementary Table 5).

Because hypoxia inducible factor (HIF) is important in regulating gene expression in low O_2_ conditions, we determined if previously published HIF targets (Ortiz-Barahona et al., 2010; Dengler et al., 2014) were upregulated in cells cultured under 5% O_2_. Indeed, we found that in all clusters, HIF targets that were differentially expressed in the data were almost always upregulated in nuclei from 5% O_2_ rather than in nuclei from 20% O_2_ (Supplementary Table 6). Certain HIF targets, such as *EGLN3* and *MMP2*, were upregulated in multiple clusters, whereas others, such as *ADM* and *VEGFA* were only upregulated in one cluster. The significance of these observations remains to be determined.

## Discussion

The goals of this research were two. The primary objective was to study the heterogeneity in cells differentiated by the BAP protocol and determine whether there was evidence for the presence of different TB sub-lineages within the population. A second was to determine whether the oxygen atmosphere under which the colonies had been cultured influenced the state of differentiation following eight days of exposure to BAP conditions. Since syncytium, identified by its expression of CGA and CGB, comprises a significant and increasing fraction of the differentiating colonies after about d 5 (Yabe et al., 2016), and because such cellular structures are large and fragile, they were not amenable to scRNAseq analysis. Accordingly, we chose to analyze transcripts present in isolated nuclei, where the issues of cell breakage and size are less likely to confound outcomes. snRNAseq has recently been used to examine transcripts during mouse placental labyrinth development (Marsh and Blelloch, 2020), where the evaluation of syncytial structures by single-cell procedures had proved to be difficult. It is also clear that the single-cell analysis performed on first trimester human placenta by Vento-Tormo et al. (2018) likely lacks a mature syncytioTB component (Roberts et al., 2021).

We note that the number of differentially expressed genes between O_2_ conditions was variable across clusters. While many genes were identified in syncytioTB cluster 6 (169 differentially expressed genes), only 44 were identified in syncytioTB cluster 5. These results could be due to the proportion of nuclei associated with each cluster in each condition. Investigating additional stages of differentiation would allow assessing changes in overall cell populations between oxygen conditions. Much more significant differences are expected to occur in early differentiation where high O_2_ appears to speed events (Amita et al., 2013), while low O_2_ permits later stage catch-up as observed here with hCG production (Supplementary Figure 1B).

The data have confirmed what has previously been contested, namely that the BMP4-driven differentiation of pluripotent stem cells results in complete or near-complete conversion to TB. However, a surprise from the analysis was the unexpectedly large number of well-defined clusters and, particularly, the presence of two major groupings (a and b) (Figure 1A), each containing distinct syncytioTB, and what appear to be diverse cytoTB components. One particular distinction between grouping a and b was the high expression of the long non-coding RNAs *NEAT1* and *MALAT1* in a, with the latter particularly abundant and, like *CGA*, expressed in almost every nucleus analyzed. Although cluster 9 is distinct from the two groupings, the expression of *NEAT1* and *MALAT1* in this cluster was more similar to grouping b. Other long non-coding RNAs, *H19, SPRY4*, and *HOTAIR* that, like *NEAT1* (Gremlich et al., 2014) and *MALAT1* (Tseng et al., 2009), have been linked to placental TB development and placental pathologies (Basak and Ain, 2019), were expressed relatively weakly and lacked meaningful discriminatory power to distinguish clusters.

It was clear that among the resulting clusters recognized in the Seurat analysis were ones that bore strong similarity to syncytioTB (5 and 6) and weaker similarity to extravillousTB (2 and 3) of first trimester placental TB. The enrichment for extravillousTB was non-significant in cluster 2, and there were only four genes (*PAPPA2, GCSH*, *ADAM12*, and *ASPSCR1*) contributing to the enrichment. Nevertheless, cluster 2, although clearly expressing TB markers, did not have similarities to any other placenta cell populations (Figure 3B). Other clusters, for example 4 and 1, that clearly did not conform strongly to either of these two sub-lineages but were sandwiched between them in the Seurat plots (Figure 1A), also expressed classical human TB markers and likely represented forms of cytoTB. We suspect that the upper regions of these two putative cytoTB clusters represent cells providing a source of syncytioTB and that their lower regions are precursors of extravillousTB. Even the more enigmatic clusters, 7, 8, and 9, display multiple TB markers, but what cell types they represent in the tissue culture colonies from which they were derived remains as yet unclear. Even within the clusters, there is additional heterogeneity. For example, the lower tip of cluster 3 and cluster 9 uniquely express *SOX2* (Supplementary Figure 8), suggesting a shared origin. In scRNAseq studies on human placentas weeks 8 and 24 of gestation (Liu et al., 2018), in first trimester (weeks 6–11) material (Suryawanshi et al., 2018), and even in blastocyst-like structures engineered from reprogrammed fibroblasts (Liu et al., 2021), it also has become clear that there are multiple distinguishable populations of cytoTBs. In the study of Liu et al. there were also three extravillousTB types (Liu et al., 2018). Moreover, cellular phenotypes as defined in terms of transcript content changed markedly between week 8 and week 24 of gestation (Liu et al., 2018). Perhaps it should be no surprise that multiple kinds of TBs comprise these cell populations derived directly from ESCs, which we have hypothesized likely represent the very early stages of *in vivo* placentation. A planned time-course experiment beginning when differentiation is initiated should enable us to infer cluster origins and interrelationships in a more complete manner.

Our ability to define two distinct syncytioTB clusters (5 and 6) is of particular interest. Do these nuclei represent different kinds of syncytioTB or nuclei with different cytoTB origins in a single type of syncytioTB? The fact that the transcripts for the presumed fusogens, ERVW-1 and ERVV-1, were primarily marker genes for nuclei in cluster 6 and less well expressed in cluster 5 could be evidence that syncytioTB formation requires the interaction of two select populations of presumptive syncytioTBs, one of which expresses the necessary syncytins (ERVs) on their surfaces, the other possibly bearing the appropriate “receptor” factors. Similarly, the absence of *MFSD2A*, which encodes the proposed receptor for ERVFRD-1, another proposed fusogen (Roberts et al., 2021), is puzzling. Perhaps another fusion partner for ERVFRD-1 exists or syncytialization of these TBs occurs without the involvement of ERVFRD-1, which has quite low expression in these preparations of ESC-derived TB.

Another observation of note is that *CGA*, whose translation product partners with one of the CGB isoforms to form the active placental hormone hCG, is expressed in most, if not all, nuclei (Figure 2), yet the protein itself can normally be detected only in syncytioTB and what appear to be its immediate precursor syncytioTB when H1 ESCs are differentiated to syncytioTB (Amita et al., 2013; Yabe et al., 2016). There seems to be three possible explanations: (1) *CGA* mRNA is not translated in cells that are not progressing to syncytioTB; (2) the protein product CGA is highly unstable in the context of cells outside the syncytial area and in absence of expression of its partner CGB with which it forms the hCG heterodimer; (3) *CGA* transcripts are unable to exit the nucleus for translation except in syncytioTB. Gene expression changes that are primarily regulated at the protein level have been noted previously in the mouse placenta and other cell types (Abdulghani et al., 2019). In fact, a general rule is that mRNA concentrations are relatively poor guides to protein levels (Ghaemmaghami et al., 2003; Ghazalpour et al., 2011; Schwanhausser et al., 2011). A number of other highly expressed transcripts, for example those for *GABRP* and *VTCN1*, are also abundant across clusters yet their proteins are restricted to emerging syncytioTB in BAP colonies. They are also expressed in placenta where they are localized to villous syncytioTB, primarily to the first trimester of pregnancy (Karvas et al., 2020). Interestingly, expression of the CD274 molecule, perhaps better known as B7-H1 or programmed cell death 1 ligand 1, also appears to be controlled at the translational level in placental TB cells (Holets et al., 2009). Both VTCN1 and CD274 bind to receptors on lymphocytes. In cancer cells and possibly in TB they are considered regulators of immune tolerance (Holets et al., 2006; Schust et al., 2021).

In summary, the BAP model, which we have proposed represents TB associated with the implanting conceptus, reveals a relatively complex picture of TB emergence, including the appearance of at least two kinds of syncytioTB nuclei plus multiple cytoTB populations. Conducting similar analyses at earlier time points should elucidate how these lineages arose and diverged and perhaps provide insights into what occurs during the very earliest stages of human embryonic development. Such studies may also be revealing about how syncytioTB arises, mechanisms of cell fusion, and the possible roles of the non-coding RNAs *MALAT1* and *NEAT1*, and various transcription factors in directing events.

## Data Availability Statement

The datasets presented in this study can be found in online repositories. The names of the repository/repositories and accession number(s) can be found below: https://www.ncbi.nlm.nih.gov/geo/, GSE171768. The GitHub repository documenting all the analyses is available at https://github.com/Tuteja-Lab/BAP.hESC.d8_.

## Author Contributions

TK, AS, JZ, NB, and TE performed the experiments. RR and TE conceived the project. RR, TE, TK, AS, and GT designed the study and were responsible for data interpretation. RR, TK, AS, and GT wrote the manuscript with contributions from all authors. RR, TE, GT, and DS sponsored the project through grants. All authors contributed to the article and approved the submitted version.

## Conflict of Interest

The authors declare that the research was conducted in the absence of any commercial or financial relationships that could be construed as a potential conflict of interest.

## Abbreviations

ANOVA: Analysis of variance
AP: **A**83–01, **P**D1730
BAP: **B**one morphogenic protein-4, **A**83–01, **P**D1730
bp: Base pair
CytoTB: Cytotrophoblast
GEMs: Gel Bead-in-Emulsion
hESCS: Human embryonic stem cells
UMAP: Uniform manifold approximation and projection
UMIs: Unique Molecular Identifiers
KOSR: Knock-out serum replacement
MEFs: Mouse embryonic fibroblast
NWRB: Nuclear wash and resuspension buffer
PCA: Principle component analyses
scRNAseq: Single cell RNA sequence
SNN: Shared nearest neighbor
snRNA: Single nucleus RNA sequence
SyncytioTB: Syncytiotrophoblast
TB: Trophoblast
t-SNE: t-distributed stochastic neighbor embedding
VST: Variance stabilizing transformation.
