# Supplementary material for: Single Nucleus RNA Sequence (snRNAseq) Analysis of the Spectrum of Trophoblast Lineages Generated From Human Pluripotent Stem Cells in vitro
Source: Front Cell Dev Biol. 2021 Jul 21;9:695248. doi: 10.3389/fcell.2021.695248 (PMC8334858; doi:10.3389/fcell.2021.695248)
Supplement: Supplementary file 1 [file Data_Sheet_1.PDF]

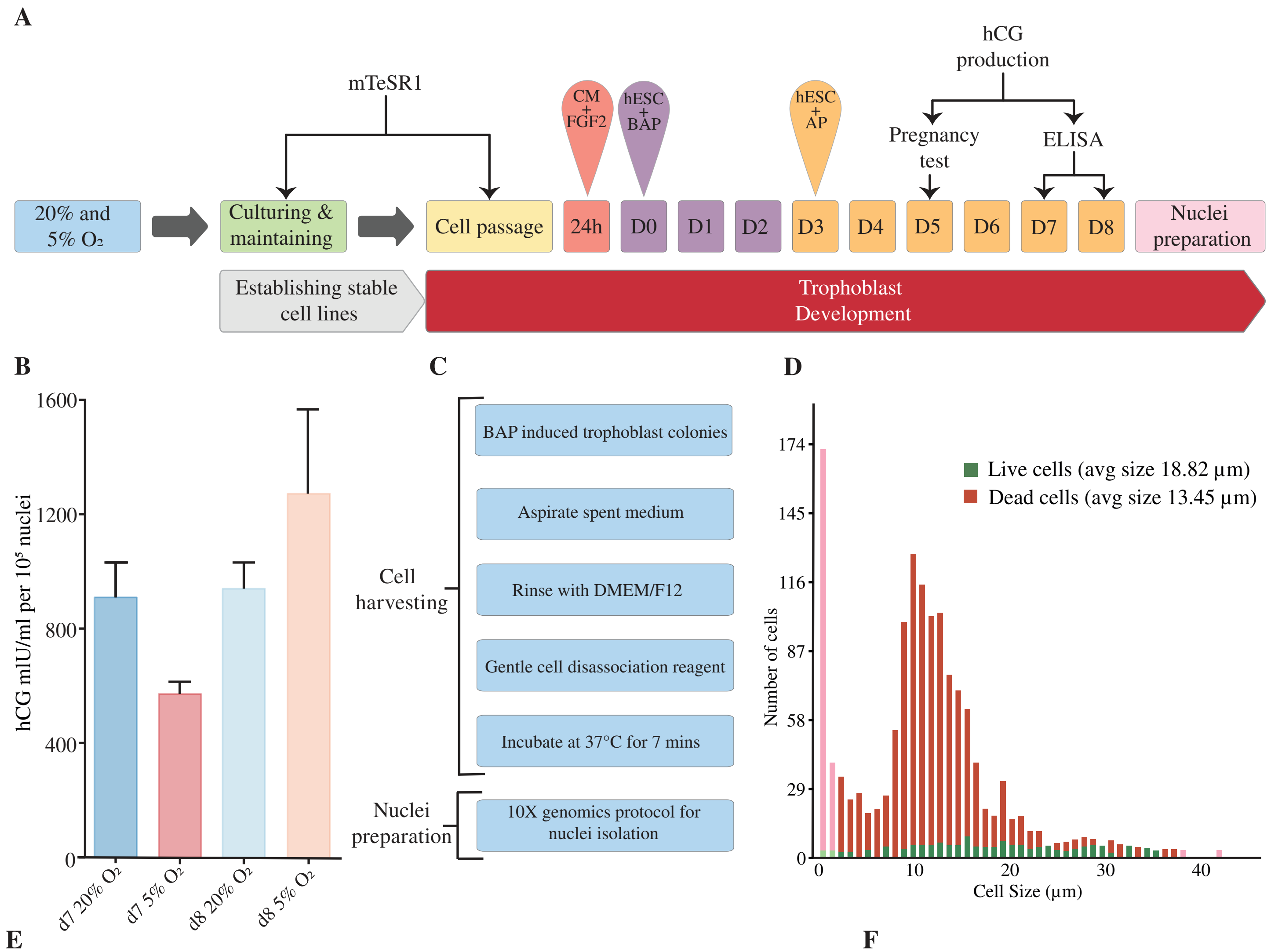

E

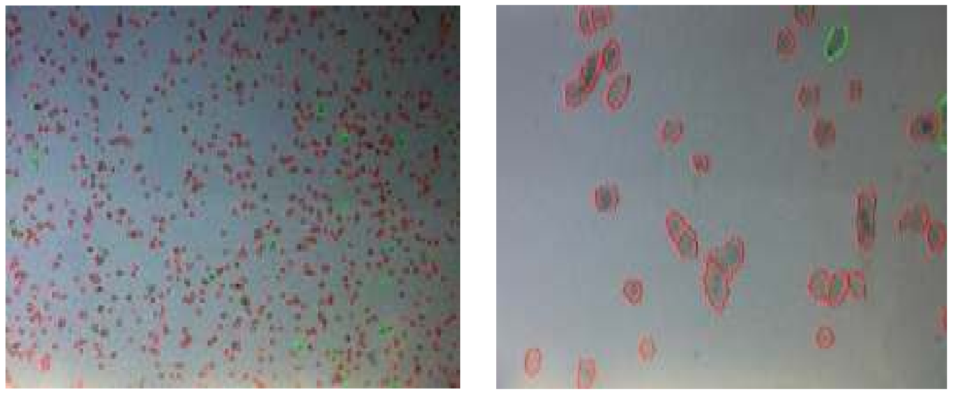

F

Quality check of isolated nuclei			
condition	replicate	intact cells (%)	conc. (nuceli/μl)
20% O_2	1	5	1975
	2	3	1650
5% O_2	1	3	1785
	2	3	1550

Supplementary Figure 1. Nuclei isolation from trophoblast. (A) Strategy for BAP directed trophoblast development. Human embryonic stem cells (hESCs) (H1) previously acclimated and maintained at 20% O_2 and 5% O_2 were thawed and passaged on mTeSR1 medium several times to established stable lines of their respective O_2 concentration conditions. The day following passaging of both 20% O_2 and 5% O_2, mTeSR1 medium was replaced with FGF2 (4 ng/μL) supplemented conditioned medium (mouse embryonic fibroblast (MEF)-conditioned DMEM/F12 with knockout serum replacement (KOSR) medium). The next day, to induce trophoblast differentiation, the conditioned medium was replaced with BMP4 (10 ng/mL), A83-01 (1 μM), and PD173074 (0.1 μM) supplemented DMEM/F12/KOSR (BAP/DMEM/F12/KOSR) medium for 3 days (BAP treatment) and the same medium without BMP4 (AP treatment) until day 8. (B) Production of human chorionic gonadotropin (hCG). hCG production was examined with pregnancy strips (not shown) on day 5

and ELISA; conducted at day 7 and day 8 for BAP directed trophoblast cells colonies cultured on both 20% O_2 and 5% O_2 conditions. 1-way ANOVA analysis with GraphPad Prism was performed which did not find any significant differences between the values. (C) Schematic of nuclei isolation (for details see methods). (D) Representative micrograph showing the average size of intact cells (green colored bars) and isolated nuclei (red colored bars). Bars in light green and pink colors indicate the cell debris or other non-cell/nuclei particles with < 3 µm diameter, being detected and ignored for counting by the Countess II automated cell counter (recreated from the image generated by the Countess II automated cell counter). (E) Representative images showing isolated nuclei. Full view (left panel) and enlarged view (right panel). Scale bar, 48 µm. Green and red circles indicate intact cells and isolated nuclei respectively. (F) Percentage of intact cells and concentration of isolated nuclei from each replicate of 20% O_2 and 5% O_2 conditions. CM, conditioned medium; FGF2, fibroblast growth factor-2; BAP, (B; Bone morphogentic protein-4 (BMP4); A, A8301 (Activin/Nodal inhibitor); P, PD173074; AP, A, A8301 (Activin/Nodal inhibitor); P, PD173074; hCG, human chorionic gonadotropin; D, day; h, hours.

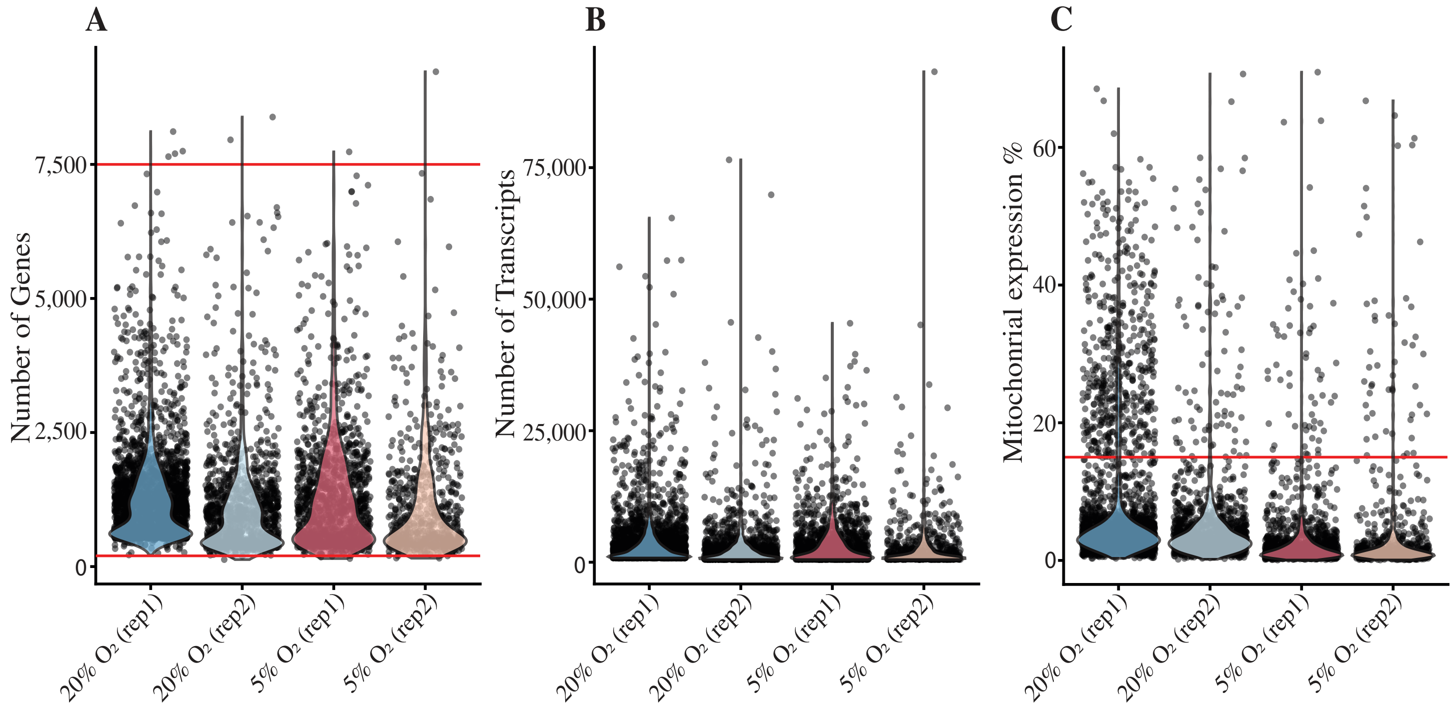

Supplementary Figure 2. Quality assessment for the snRNAseq datasets. (A) number of unique genes detected in each nucleus across samples. Red lines at 200 and 7,500 shows the cut-off for minimum and maximum number of genes used in the analyses. (B) Total number of molecules (transcripts) detected within a nucleus, across samples. (C) Mitochondrial expression (percent) in each nucleus across samples. Red line at 15% indicates the threshold used for filtering off nuclei for the analyses. Each dot represents a nucleus. 20% O_2, nuclei obtained from the cells cultured under 20% O_2; 5% O_2, nuclei obtained from the cells cultured under 5% O_2; rep, replicate.

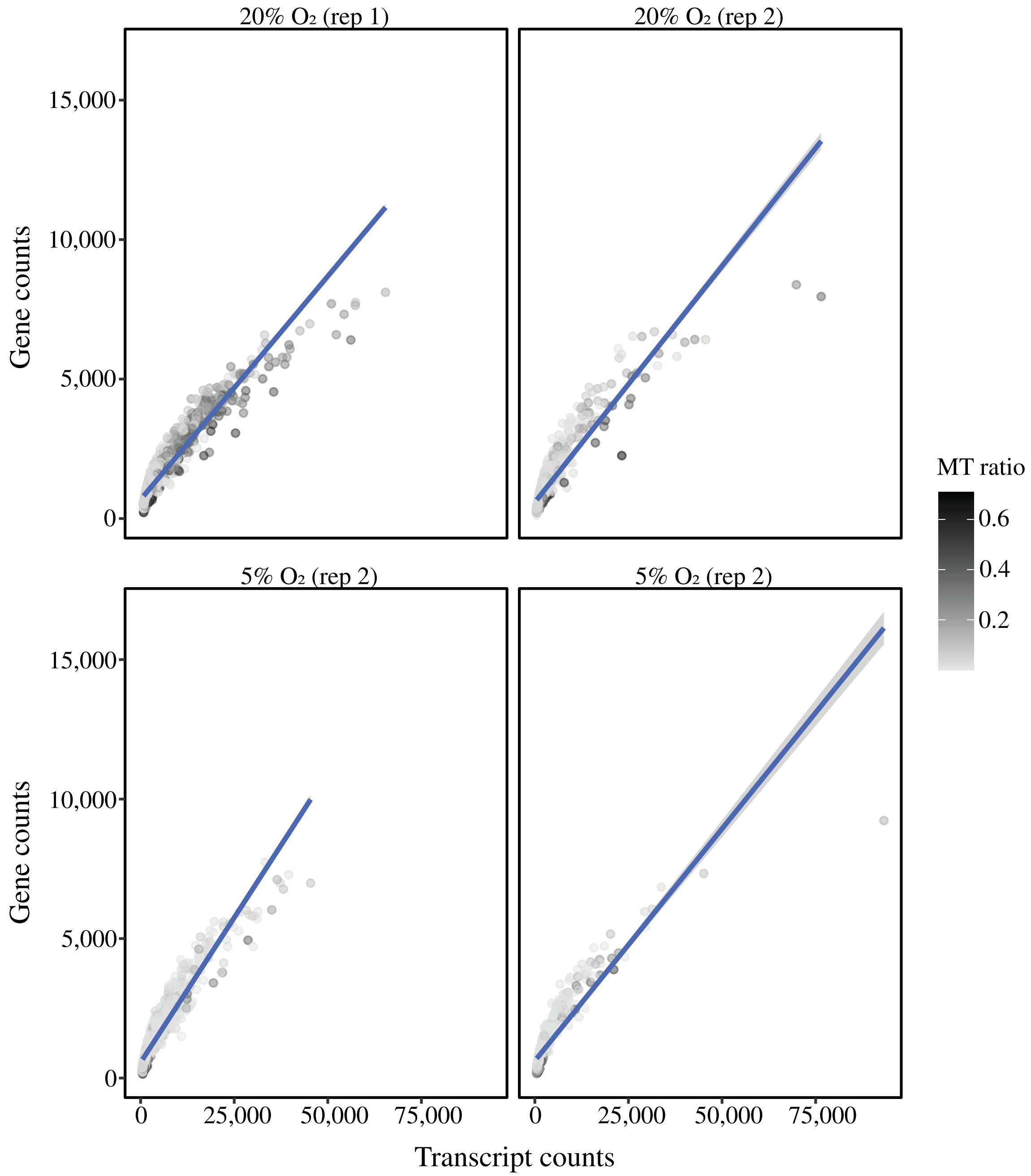

Supplementary Figure 3. Relationship between the total molecules detected (transcripts) versus total genes detected across the samples. Each nucleus is represented as a dot, with the color intensity representing the mitochondrial read ratio in that nucleus. 20% O_2, nuclei obtained from the cells cultured under 20% O_2; 5% O_2, nuclei obtained from the cells cultured under 5% O_2; rep, replicate; MT, mitochondria.

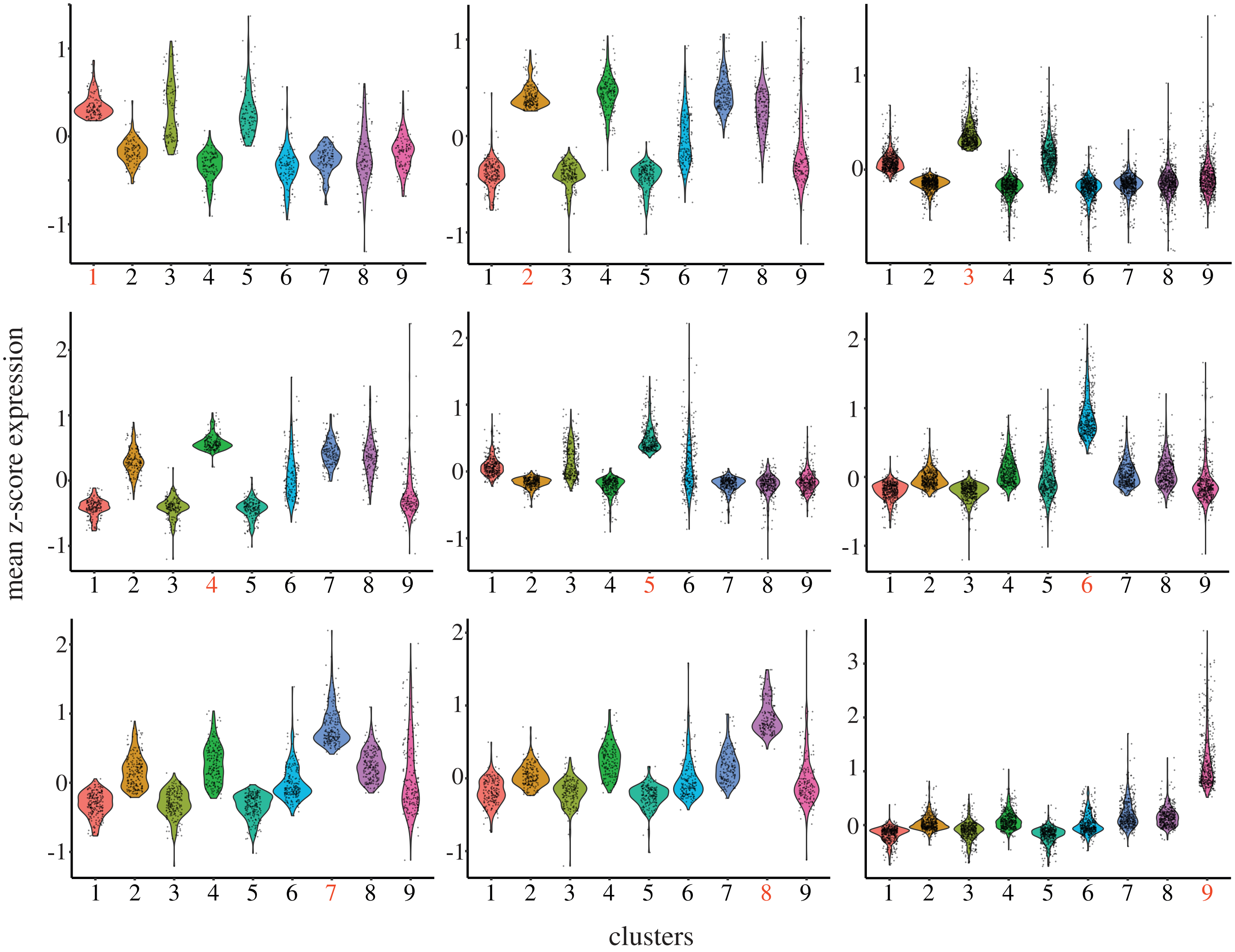

Supplementary Figure 4. Cell-specific markers reveal higher expression in their respective cluster. The number in red (x-axis) indicates the cluster to which these cell-specific markers belong to. The expression levels are standardized using the z-score average for comparing a subset of markers across clusters.

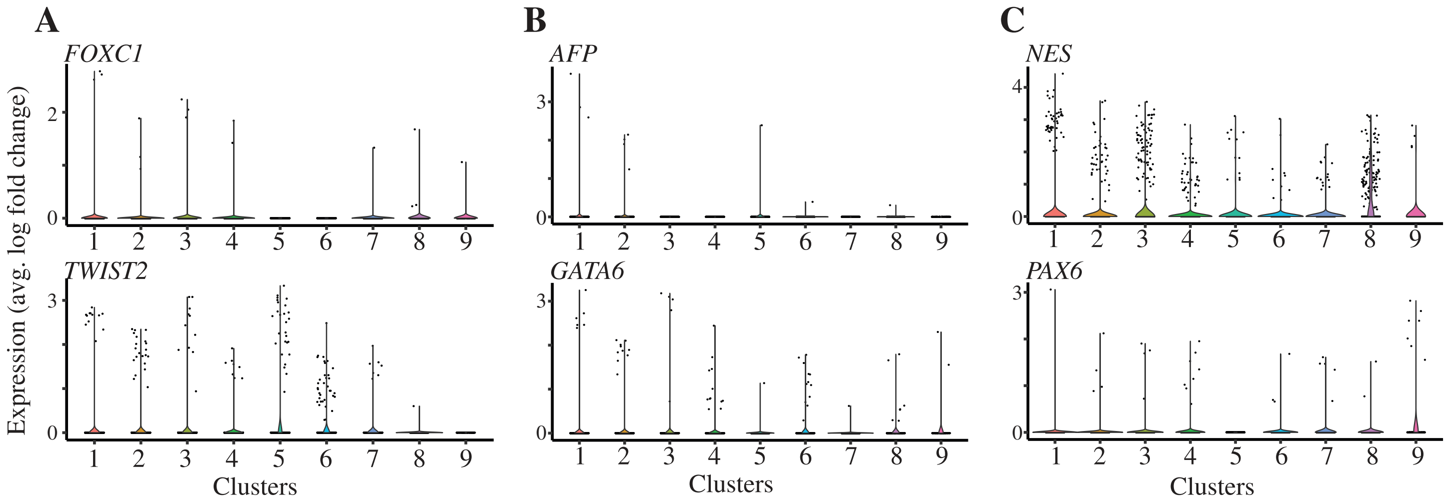

Supplementary Figure 5. Germinal layer markers were least expressed. Violin plots showing low expression levels for (A) mesoderm (B) endoderm, and (C) ectoderm genes.

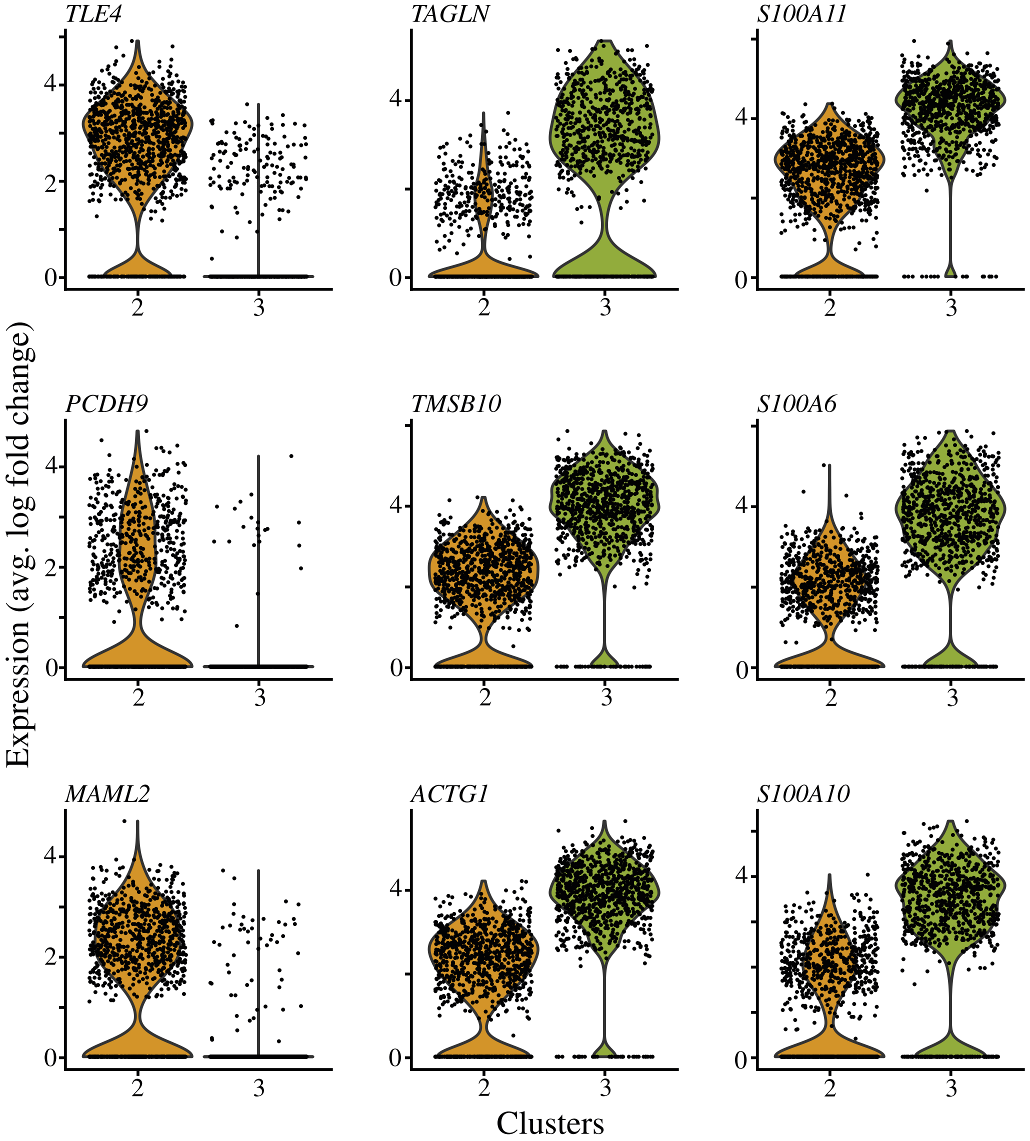

Supplementary Figure 6. Differential expression of extravillousTB markers. Genes that are differentially expressed in extravillousTB clusters 2 and 3 are shown.

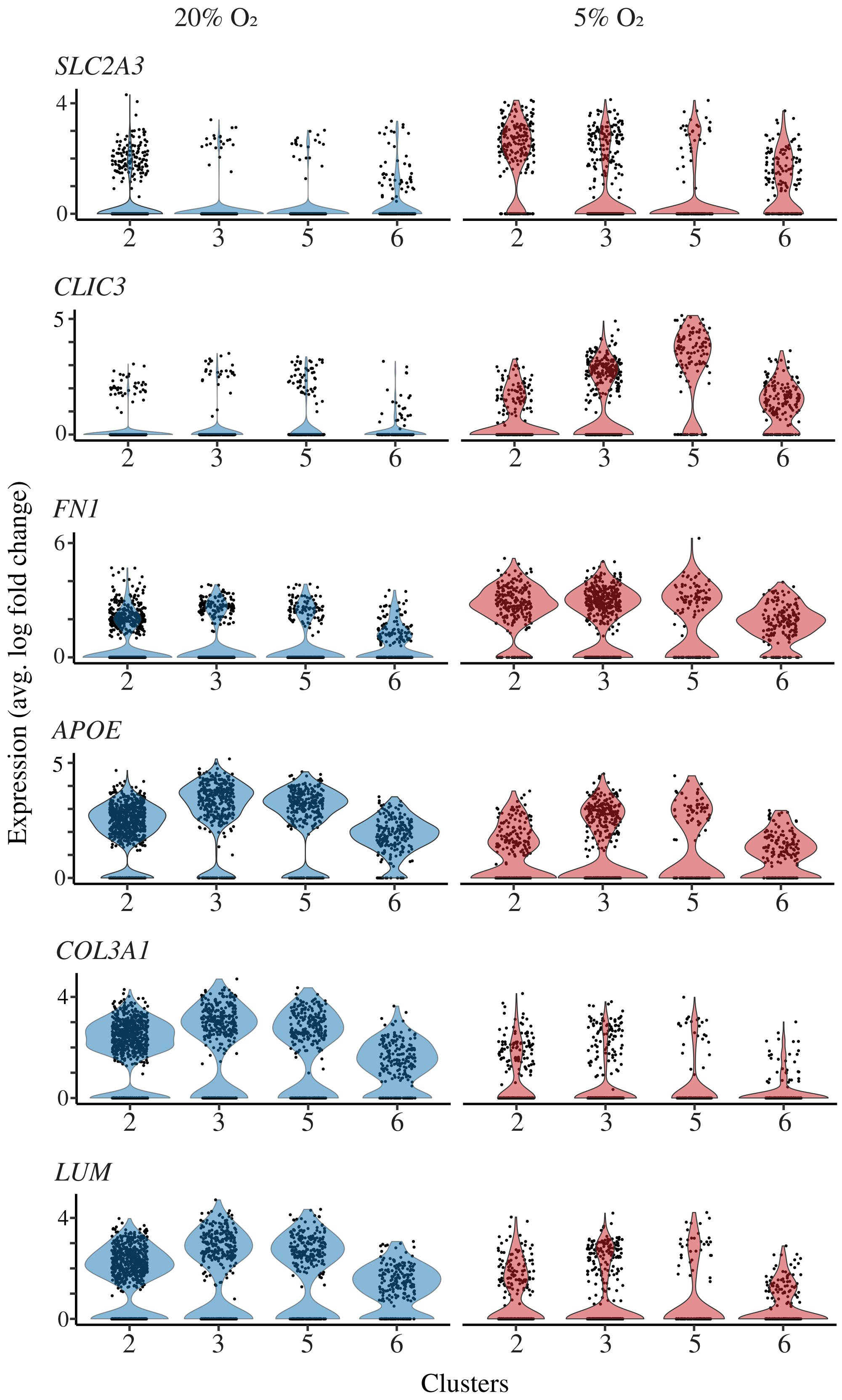

Supplementary Figure 7. Differential expression of selected genes across treatments shown as violin plots. The expression of SLC2A3, CLIC3, and FN1 is higher in nuclei obtained from the cells cultured under 5% O_2 concentration conditions. In contrast, genes APOE, COL3A1, and LUM are higher in nuclei obtained from the cells cultured under 20% O_2 concentration conditions.

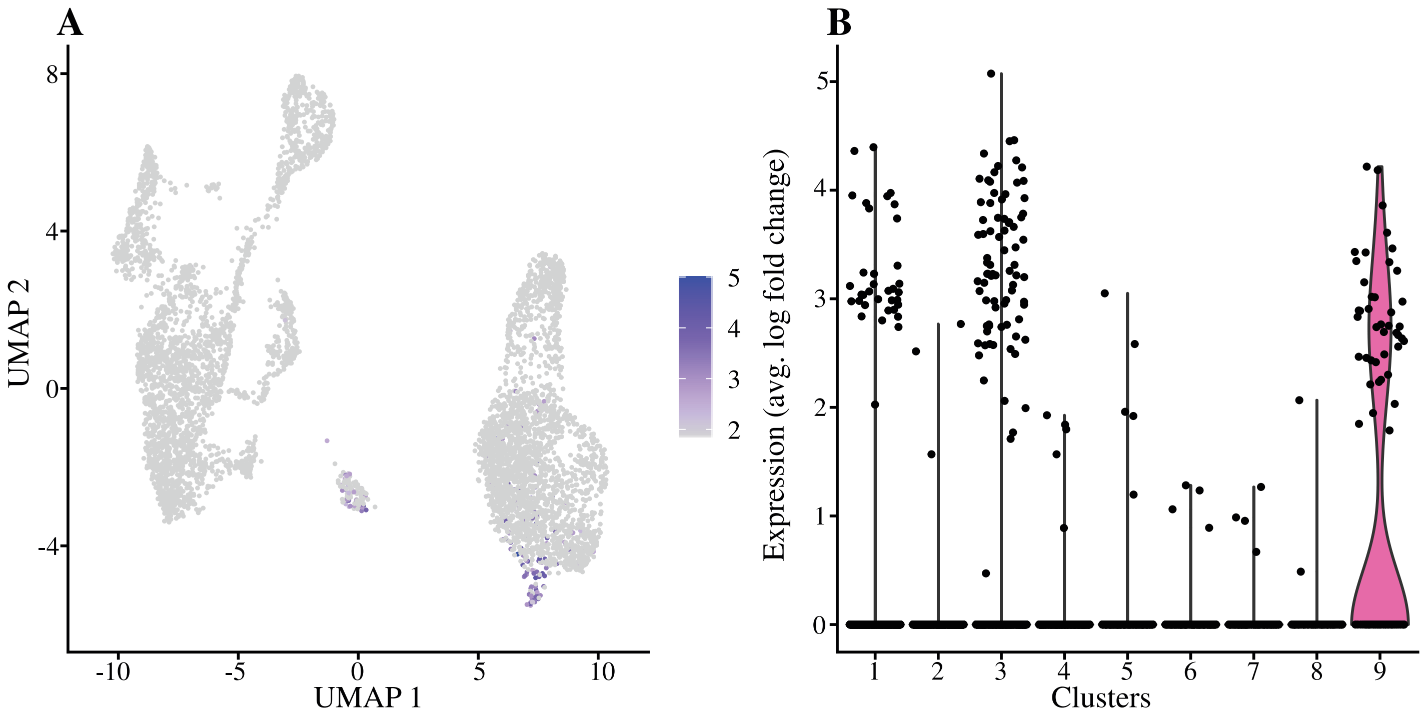

Supplementary Figure 8. Heterogenous expression of SOX2. (A) SOX2 localization in the dimensionality reduction plot. (B) SOX2 expression across clusters.
